# Managing toxicities and optimal dosing of targeted drugs in advanced kidney cancer

**DOI:** 10.3747/co.v16i0.402

**Published:** 2009-05

**Authors:** B. Seruga, H.K. Gan, J.J. Knox

**Keywords:** Sunitinib, sorafenib, temsirolimus, toxicity, side effects, management

## Abstract

The toxicities of new, targeted drugs may diminish their effectiveness in advanced kidney cancer if those toxicities are not recognized and properly addressed early in patient treatment. Most of the drug-related toxicities in advanced kidney cancer are manageable with supportive care, obviating a need for long interruptions, dose reductions, or permanent discontinuation of the treatment.

## 1. INTRODUCTION

Sunitinib, sorafenib, temsirolimus, and bevacizumab are new drugs used in treatment of advanced kidney cancer 1–4, together with another emerging agent, everolimus 5. By targeting healthy tissues, these drugs cause toxicities that can lead to delay, dose reduction, or discontinuation of the drug treatment. When such disruptions occur early on, the potential benefit to the patient of these new agents is lost.

The use of targeted agents in a less selected patient population outside of clinical trials can certainly be challenging, but experience to date shows that most of the toxicities associated with the new agents are manageable. The data about the toxicities of sunitinib and sorafenib outside of clinical trials in expanded-access programs are reassuring 6,7. In the present review, we describe the recommended doses of the new drugs and the management of common toxicities of drugs used in advanced kidney cancer.

## 2. INHIBITORS OF KINASES: SUNITINIB AND SORAFENIB

Sunitinib and sorafenib target tyrosine kinases, including vascular endothelial growth factor receptors (vegfrs) 8. The humanized monoclonal antibody bevacizumab inhibits vascular endothelial growth factor A (vegfa), but is not discussed in this review. (It is not approved for use in advanced kidney cancer in Canada.)

### 2.1 Recommended Treatment Schedules and Dose Adjustments

#### 2.1.1 Sunitinib

From the drug label 9, the starting dose for sunitinib is 50 mg orally once daily with or without food, in a 4-weeks-on, 2-weeks-off regimen. Standard dose modification in 12.5-mg steps is recommended. If more than 2 dose reductions are required, discontinuation of therapy should be considered. Recovery to acceptable levels of toxicity must occur to allow sunitinib continuation. Re-escalation to the previous dose schedule can be considered in the absence of grade 3 or greater hematologic toxicity, or in the absence of grade 2 or greater non-hematologic toxicity. The drug label recommends permanent discontinuation of sunitinib in the rare case of congestive heart failure, pancreatitis, or hepatic failure.

Clinical studies excluded patients with a serum creatinine in excess of 2 times the upper limit of normal (uln), and so caution is recommended in the presence of severe kidney impairment. No dose adjustment is required for Child–Pugh class A or B hepatic impairment, but sunitinib has not been studied in severe (Child–Pugh class C) liver impairment. Clinical studies excluded patients with aminotransferase levels in excess of 2.5 times the uln or, if a result of liver metastases, in excess of 5 times the uln 9.

Based mostly on clinical experience and very limited data from early-phase clinical trials 10,11, alternative regimens of sunitinib might be considered: either a 2-weeks-on, 1-week-off regimen, or continuous daily dosing with 37.5 mg. However, efficacy confirmation for these latter regimens is lacking.

#### 2.1.2 Sorafenib

The recommended dose of sorafenib is 400 mg orally twice daily without food (at least 1 hour before or 2 hours after a meal). When dose reduction is required, the dose may be reduced to 400 mg once daily, and further to 400 mg once every other day. An alternative, more gradual reduction is to use one dose of 200 mg while the second dose is maintained at 400 mg (600 mg daily total); reduction to 400 mg daily can then follow if toxicity persists.

Permanent discontinuation should be considered in the unlikely events of bleeding that requires medical intervention or of gastrointestinal perforation. Temporary interruption should be considered in patients undergoing major surgical procedures and in whom cardiac ischemia or infarction occur; in the latter setting, permanent discontinuation can be considered. No dose adjustment is necessary in patients with kidney impairment who are not undergoing dialysis (patients on dialysis have not been studied). Mild and moderate (Child–Pugh A and B) liver impairment may reduce plasma levels of sorafenib, but the optimal dose of sorafenib in hepatic impairment has not been established 12.

### 2.2 Drug Metabolism and Dose Modifications

Sunitinib and sorafenib are metabolized primarily by cytochrome P450 (CYP) 3A4 in the liver, and sorafenib additionally undergoes glucuronidation by uridine glucuronyl transferase 1A9 and by being a competitive inhibitor of CYP2B6 and CYP2C8 isozymes 9,12. Concomitant use of medications that substantially affect these metabolizing enzymes should be avoided (Tables i and ii). The dose of sunitinib should be reduced to a minimum of 37.5 mg if strong CYP3A4 inhibitors must be co-administered and possibly increased to a maximum of 87.5 mg if strong CYP3A4 inducers must be co-administered (Table i). Sorafenib seems not to have any clinically significant pharmacokinetic interactions with strong CYP3A4 inhibitors, but if a strong CYP3A4 inducer must be co-administered, an increase of the sorafenib dose should be considered 12.

### 2.3 Common Toxicities

The most commonly reported clinical toxicities are constitutional, gastrointestinal, hypertensive, and skin-related; the most common laboratory toxicities are lymphopenia, neutropenia, thrombocytopenia, anemia, elevated lipase, and hypophosphatemia 1,2. In pivotal randomized clinical trials (rcts), severe—that is, grades 3 and 4—toxicities were reported in two thirds of patients on sunitinib 1 and in more than one third of patients on sorafenib 2.

## 3. TEMSIROLIMUS

Temsirolimus is a potent, highly specific inhibitor of the mammalian target of rapamycin (mtor). It inhibits cell proliferation, cell growth, survival pathways, and tumour angiogenesis 13. Everolimus (RAD001) is an orally administered mtor inhibitor currently under review by Health Canada.

### 3.1 Recommended Treatment Schedule and Dose Adjustments

The recommended dose of temsirolimus in advanced kidney cancer is a flat dose of 25 mg infused over 30–60 minutes once weekly. Before each administration of temsirolimus, patients should receive intravenous (IV) H_1_ antihistamine—for example, IV diphenhydramine 25–50 mg 30 minutes before temsirolimus. If hypersensitivity occurs, the infusion should be stopped for at least 30–60 minutes and resumed at a slower rate approximately 30 minutes after IV administration of an H_2_ antihistamine—for example, IV famotidine 20 mg or ranitidine 50 mg 14. If a severe hypersensitivity reaction occurs, re-challenge with temsirolimus is contraindicated.

Temsirolimus should be held in the presence of an absolute neutrophil count below 1000/μL, a platelet count below 75,000/μL, or any other grade 3 or greater toxicity. Once toxic effects have resolved to grade 2 or lesser, temsirolimus can be restarted at 20 mg or 15 mg weekly, but no lower. No data on dosing in patients with kidney and hepatic impairment are available 14.

### 3.2 Drug Metabolism and Dose Modifications

Temsirolimus is metabolized by CYP3A4, and strong inducers and inhibitors should not be co-administered (Table i). If a strong CYP3A4 inhibitor or inducer must be co-administered, the dose of temsirolimus should be reduced to 12.5 mg weekly or cautiously increased to 50 mg weekly respectively. If the strong CYP3A4 inhibitor is discontinued, a 1-week washout period should elapse before temsirolimus is re-instituted at the dose level previously used 14.

### 3.3 Common Toxicities

The most common clinical toxicities of temsirolimus are constitutional and skin-related; the most common laboratory toxicities are hyperglycemia, hypertriglyceridemia, hypercholesterolemia, hypophosphatemia, and hematologic toxicities. In a pivotal rct, the most common severe toxicities were anemia (13%), hyperglycemia (9%), asthenia (8%), and hypertriglyceridemia (3%) 15. Rare but fatal cases of nonspecific interstitial pneumonitis have been reported in phase i and ii trials in patients with advanced kidney cancer on temsirolimus 16. Most temsirolimus-related toxicities can be managed medically or with supportive care without a need for interruption or dose reduction.

## 4. MANAGEMENT OF TOXICITIES

### 4.1 Fatigue

Fatigue has been reported in 51% and 37% of patients on sunitinib and sorafenib respectively; severe fatigue occurred in 5%–7% of patients 1,2. In a rct of sorafenib, patients with advanced kidney cancer on sorafenib did not experience significantly more fatigue than did patients on placebo 2.

Fatigue may be associated with hypothyroidism, anemia, depression, or dehydration, all of which can be addressed. Thyroid hormone replacement therapy benefited 50% of patients who developed overt hypothyroidism on sunitinib 17, indicating that other mechanisms causing fatigue are at play in these patients. Physical activity is effective in the management of cancer-related fatigue 18, and patients should be counselled to do some light exercise, if appropriate. In severe fatigue, treatment should be interrupted and the dose reduced (Table iii).

### 4.2 Gastrointestinal Toxicity

#### 4.2.1 Diarrhea

In rcts, diarrhea occurred in 53% and 43% of patients on sunitinib and sorafenib respectively; severe diarrhea occurred in 2%–5% of patients 1,2.

Mild diarrhea (an increase of up to 6 stools daily over baseline) can be managed by oral hydration and antidiarrheal agents (for example, loperamide or diphenoxylate) alone. In severe diarrhea, patient should be hospitalized for IV hydration; treatment with sunitinib and sorafenib should be discontinued until diarrhea is grade 1 or stools have returned to baseline, when treatment can be restarted at a lower dose. During an episode of diarrhea patients should be counselled to avoid the use of stool softeners, laxatives, antacids, and a fibre-rich diet. Other causes of diarrhea, such as antibiotics, infectious gastroenteritis, partial intestinal obstruction, and radiation-induced enteritis should be excluded (Table iii).

#### 4.2.2 Nausea and Vomiting

Nausea and vomiting occurred, respectively, in 44% and 24% of patients on sunitinib and in 23% and 16% on sorafenib; severe nausea and vomiting occurred in 6% of patients only 1,2.

Common antiemetics can be used, including prophylactically, to relieve nausea and vomiting. Antiemetics such as haloperidol and 5-hydroxytryptamine3 antagonists can be associated with QT interval prolongation 19 and should be used cautiously in combination with sunitinib (see “Cardiotoxicity” later in this article).

Any constipation should be corrected (Table iii).

### 4.3 Skin Toxicity

Skin toxicity typically occurs in the first few weeks of treatment with sunitinib and sorafenib 20,21. Various skin changes may be observed, including hand–foot syndrome (hfs).

The most clinically significant skin toxicity, hfs, may lead to dose modification or discontinuation of treatment. Discontinuation of treatment in skin toxicities should be considered after the fourth occurrence of a grade 2 skin toxicity or the third occurrence of a grade 3 skin toxicity 12.

In a pooled analysis, hfs occurred in 19% (5% severe) of patients on sunitinib 22. No reports of hfs have been seen with temsirolimus. Pre-existing hyperkeratosis confers a predisposition for hfs, and preventive manicure and pedicure are recommended. Topical treatment with keratolytics such as urea 40% cream, tazarotene 0.1%, or topical fluorouracil showed a beneficial effect in a small series of patients with hfs induced by sunitinib or sorafenib 23. Moisturizers, shock absorbers, and topical corticosteroids have been found useful by many. Patients should be encouraged to avoid tight-fitting shoes and friction and to apply cream containing urea to the hands and feet daily throughout therapy. In the cases of severe HFS, treatment should be interrupted until recovery to grade 1, and the dose at resumption should be reduced (Table iii).

Skin rash associated with sunitinib, sorafenib, and temsirolimus usually presents as low-grade maculopapular or seborrheic dermatitis 15,24 that rarely requires treatment interruption or dose reduction. Urea-containing lotions may be helpful if the skin is very dry. The dermatitis can be managed with the use of fragrance-free moisturizers and topical clindamycin or corticosteroids (hydrocortisone 1% cream, for instance) 15,24. In more severe cases, systemic antibiotics (for example, orally administered doxycycline) can be used. Seborrheic-like rash with scaly areas can be treated with topical antifungals or steroids. Often, patients can attempt a re-escalation of the drug if a lower dose has been well tolerated for a period of time (Table iii).

### 4.4 Hypothyroidism

In observational studies with sunitinib, up to 85% of patients developed biochemical hypothyroidism, and approximately one quarter of all patients showed signs or symptoms of hypothyroidism that needed treatment 17,25. In contrast, in one observational study with sorafenib, biochemical hypothyroidism occurred in 18% of patients, and only 3% of patients had clinically significant hypothyroidism 26. Sunitinib-induced hypothyroidism may develop in the first few weeks after initiation of treatment and can quickly become severe 17,27. Rarely, hyperthyroidism may precede development of hypothyroidism in patients on sunitinib 9.

With both sunitinib and sorafenib, thyroid-stimulating hormone should be measured at baseline. The measurement should be repeated every 2 months with sunitinib, but only if symptoms and signs of hypothyroidism occur with sorafenib. The current recommendation is that overt and subclinical hypothyroidism should be treated 28. With recognition and proper treatment of hypothyroidism, no dose modification of sunitinib and sorafenib should be required (Table iii).

### 4.5 Hypertension

In two recent meta-analyses of prospective clinical trials, 21% (7% severe) of patients on sunitinib and 23% (6% severe) of patients on sorafenib developed arterial hypertension 29,30. Untreated arterial hypertension may be important in the exacerbation of myocardial damage 31. This hypothesis is supported by animal studies, which show that inhibition of vegfr signalling promotes transition from compensatory cardiac hypertrophy to heart failure in response to pressure overload 32,33.

Patients receiving sunitinib or sorafenib should be monitored closely for hypertension, which should be treated if necessary. Patients should be encouraged to monitor and record blood pressure daily at home. The objective of treatment is to normalize blood pressure (resting level below 140/90 mmHg). Standard hypertensive therapies are recommended 34; vasodilatators appear to be the most effective. Optimal choices are drugs not metabolized by the CYP system in the liver—for example, lisinopril and quinapril, temisartan and valsartan, atenolol and hydrochlorothiazide 35. Antihypertensive drugs that are substrates of CYP3A4—for example, enalapril, losartan, nifedipine, and amlodipine—are not ideal, but can be used cautiously. The CYP3A4 inhibitors such as verapamil and diltiazem should be avoided 35. In severe hypertension (above 200 mmHg systolic or above 110 mmHg diastolic), treatment should be temporarily interrupted until hypertension is controlled; it can then be restarted at a lower dose. Uncontrolled hypertension should be controlled before sunitinib or sorafenib is started. Patients with known hypertension will likely need assessment or dose adjustments to their antihypertensive drugs, or both. With proper management of hypertension, it is unlikely that interruption, dose reduction, or discontinuation of treatment will be required (Table iii).

### 4.6 Cardiotoxicity

Recent small observational studies showed symptomatic cardiac disease in 15%–18% of patients treated with sunitinib or sorafenib 36,37. Ongoing adjuvant trials will provide a definitive answer about the cardiotoxicity of these drugs; however, in everyday clinical practice, caution is already recommend concerning this possible toxicity. Sunitinib prolongs the QT interval in a dose-dependent manner, an effect that can occasionally lead to ventricular arrhythmias (less than 1% of cases) 9.

Baseline and periodic assessment of electrocardiogram and left ventricular ejection fraction (lvef) should be considered in patients receiving sunitinib or sorafenib, especially in those with a history of heart disease. Every patient with new symptoms of peripheral edema, dyspnea, chest pain, or dizziness (although these symptoms are common in progressing cancer *per se*) should be assessed for cardiac disease. In the presence of congestive heat failure, sunitinib and sorafenib should be discontinued, and appropriate standard therapy for heart disease should be started. The dose of sunitinib or sorafenib should be interrupted or reduced in patients without clinical symptoms of congestive heart failure but with a lvef of less than 50% or more than 20% below baseline 9. Sunitinib should be used cautiously in patients with a known history of QT prolongation, in those who are using CYP3A4 inhibitors or drugs that are known to prolong QT, and in those with electrolyte disturbances 9 (Table iii).

### 4.7 Interstitial Pneumonitis

Interstitial pneumonitis is a potentially life-threatening toxicity of temsirolimus, and patients, especially those with a history of lung disease, should be monitored for it. In patients with radiographic changes of interstitial pneumonitis but no symptoms (for example, dry cough, dyspnea, fever), treatment with temsirolimus can continue, but these patients should be followed closely. In patients with symptoms of interstitial pneumonitis, lung imaging and pulmonary function tests should be performed. Where mild symptoms are present, temsirolimus may be temporarily interrupted until symptoms resolve. In cases with severe symptoms, temsirolimus should be discontinued, and treatment with high-dose steroids (for example, 1 mg/kg prednisone) should be started. Tapering of the steroid dose should be considered if gradual improvement in symptoms and pulmonary function tests occur 15,16 (Table iii).

### 4.8 Hyperglycemia

Fasting blood sugar should be tested before and regularly during treatment with temsirolimus 15. Hyperglycemia may manifest as excessive thirst, increased urination, and blurred vision; if untreated, it may lead to coma. Management of hyperglycemia may include diet, orally administered agents for glycemic control (metformin, for instance), and insulin, if required 15 (Table iii).

### 4.9 Hyperlipidemia

Serum cholesterol and triglycerides should be measured at baseline and regularly every 1–2 months during treatment with temsirolimus. If levels are elevated, a diet with reduced saturated fats and addition of lipid-lowering agents could be considered. Pravastatin is the only statin that is not a substrate of CYP3A4; it is the safest option in combination with temsirolimus 35. However, use of lipid-lowering medications may not be practical in this advanced cancer population. When triglyceride levels exceed 1000 mg/dL (11.3 mmol/L), the risk of triglyceridemia-induced pancreatitis is high, and in patients without hepatic and renal insufficiency, fibrates (for example, gemfibrozil and fenofibrate) can be considered 38; otherwise, interruption of treatment is recommended (Table iii).

### 4.10 Hematologic and Other Laboratory Toxicities

A complete blood count should be obtained at the beginning of each treatment cycle. If grades 1 and 2 hematologic toxicities (hemoglobin not lower than 8 g/dL, leucocyte count not lower than 2000/mm^3^, and platelet count not lower than 50,000/mm^3^) are present, treatment can be continued at the same dose level. If grade 3 toxicity is present, the dose should be held until toxicity reaches grade 2 or lower, at which time treatment can be resumed at the same dose. If grade 4 toxicity is present, the dose should be held until toxicity reaches grade 2 or lower, at which time treatment can be resumed at a reduced dose. For symptomatic anemia, blood transfusions without treatment interruption and dose reduction may be offered.

Serum lipase and amylase should be followed, and consumption of alcohol should be discouraged if these parameters become elevated. In rcts, clinical pancreatitis developed in only 3 patients (fewer than 1%) on sorafenib 12. A diagnosis of pancreatitis should not be made solely on the basis of abnormal laboratory values 12. If a patient develops clinical symptoms of pancreatitis, sunitinib or sorafenib should be held while the symptoms are investigated (Table iii).

Phosphate levels should be monitored before each treatment cycle, and in hypophosphatemia, oral phosphate replacement (that is, 1 tablet of sodium acid phosphate twice daily with meals) should be considered (Table iii).

## 5. CONCLUSIONS

Patients with advanced kidney cancer need to be closely monitored for the development of drug-related toxicities. Development of drug-related toxicities (even at mild and moderate levels) should be vigorously managed with supportive measures to prevent interruptions of treatment, dose reductions, and eventual development of life-threatening complications.

## Figures and Tables

**TABLE I:** Agents that interact with cytochrome P450 3A4

*Strong^a^*	*Inhibitors Moderate^b^*	*Mild^c^*	*Inducers*	
hiv antivirals (indinavir, nelfinavir, ritonavir) Clarithromycin Ketoconazole Itroconazole Nefazodone Saquinavir Telithromycin	Aprepitant Fluconazole Erythromycin Verapamil Diltiazem Grapefruit juice	Cimetidine Amiodarone Norfloxacin Voriconazole Delaviridine Fluvoxamine Gestodene Imatinib Star fruit	hiv antivirals (efavirenz, nevirapine) Barbiturates Carbamazepine Glucocorticoids Modafinil Nevirapine Oxcarbazepine	Phenobarbital Phenytoin Pioglitazone Rifabutin Rifampin Troglitazone St. John’s Wort

*Substrates*				

Macrolide antibiotics (clarithromycin, erythromycin, *not* azithromycin) Anti-arrhythmics (quinidine) Benzodiazepines (midazolam, diazepam, alprazolam, triazolam)	Immune modulators (cyclosporine, tacrolimus) hiv antivirals (indinavir, nelfinavir, ritonavir, saquinavir)	Antihistamines (astemizole, chlorpheniramine, terfenadine) Calcium channel blockers (amlodipine, diltiazem, nifedipine, verapamil)	Statins (atorvastatin, cerivastatin, lovastatin, simvastatin, *not* pravastatin) Steroids (estradiol, hydrocortisone, progesterone, testosterone)	Miscellaneous (aprepitant, ondansetron, caffeine, domperidone, haloperidol, fentanyl, lidocaine, propranolol, salmeterol, quinine)

^a^ Increase by more than a factor of 5 in plasma area under the curve (auc) value, or more than 80% decrease in clearance.

^b^ Increase by more than a factor of 2 in plasma auc value, or 50%–80% decrease in clearance.

^c^ Increase by a factor of 2 or less in plasma auc value, or less than 50% decrease in clearance.

**TABLE II:** Agents that interact with cytochrome P450 2B6 and cytochrome P450 2C8^a^

*Strong^b^*	*Inhibitors Moderate^c^*	*Mild^d^*	*Inducers*	
Gemfibrozil	Trimethoprim	Ticlopidine, montelukast, quercetin, glitazones	Rifampin	Phenobarbital

*Substrates*				

Bupropion	Cyclophosphamide	Efavirenz	Ifosfamide	Methadone
Paclitaxel	Torsemide	Amodiaquine	Cerivastatin	Repaglinide

^a^ Applicable to sorafenib only.

^b^ Increase by more than a factor of 5 in plasma area under the curve (auc) value, or more than 80% decrease in clearance.

^c^ Increase by more than a factor of 2 in plasma auc value, or 50%–80% decrease in clearance.

^d^ Increase by a factor of 2 or less in plasma auc value, or less than 50% decrease in clearance.

**TABLE III:** Summary of toxicities induced by anticancer drugs in kidney cancer, and related management strategies

*Toxicity*	*Management*
Fatigue	Exclude other possible causes of fatigue.
	Light exercise is recommended (if appropriate).
	If severe fatigue persists, interrupt treatment and reduce the dose.
Diarrhea	Exclude other possible causes of diarrhea.
	In mild diarrhea, use oral hydration and antidiarrheal agents (for example, loperamide, diphenoxylate).
	In severe diarrhea, use hospitalization and intravenous hydration; interruption of treatment with sunitinib or sorafenib is recommended. (Restore treatment when diarrhea reaches grade 1 or stools have returned to baseline.)
Nausea and vomiting	Use common oral antiemetics for treatment and prevention.
	Use haloperidol and 5-hydroxytryptamine3 antagonists with sunitinib cautiously (possible QT prolongation).
Hand–foot syndrome (hfs)	Before start of treatment, manicure and pedicure are recommended.
	Prophylactic use of cream containing urea is recommended during treatment.
	In mild hfs, use topical treatments: urea 40% cream, tazarotene 0.1%, topical fluorouracil, moisturizers, corticosteroids, shock absorbers.
	In severe hfs, treatment with sunitinib or sorafenib should be interrupted until recovery to grade 1 status, and then dose should be reduced.
Skin rash	In mild rash, use topical treatments: urea-containing lotion, fragrance-free moisturizers, topical clindamycin and corticosteroids (for example, hydrocortisone cream 1%).
	In severe rash (rare), use systemic antibiotics (for example, oral doxycycline).
	Consider dose-reduction of anticancer drugs if severe rash persists.
Hypothyroidism	Thyroid-stimulating hormone (tsh) should be measured before start of treatment with sunitinib or sorafenib.
	With sunitinib, tsh should be repeated every 2 months; with sorafenib, repeat tsh only if overt hypothyroidism occurs.
	Overt and subclinical hypothyroidism should be treated.
	Dose reduction with sunitinib and sorafenib is usually not required.
Hypertension	Resting blood pressure should be below 140/90 mmHg before and during treatment.
	Use optimal antihypertensive drugs: lisinopril, quinapril, temisartan, valsartan, atenolol, or hydrochlorothiazide [no cytochrome P450 (CYP) 3A4 interactions].
	Use enalapril, losartan, nifedipine, and amlodipine cautiously (CYP3A4 substrates).
	Avoid verapamil and diltiazem (CYP3A4 inhibitors).
	In severe hypertension (above 200 mmHg systolic, above 110 mmHg diastolic), temporarily interrupt treatment until hypertension is controlled; restart sunitinib or sorafenib at a lower dose.
Cardiotoxicity	Blood pressure should be controlled.
	Baseline and periodic assessment of electrocardiogram and left ventricular ejection fraction (lvef) should be considered in patients on sunitinib and sorafenib, especially in patients with a history of heart disease.
	Interrupt or reduce the dose of sunitinib or sorafenib in patients without clinical symptoms of congestive heart failure (chf), but with lvef below 50% or more than 20% below baseline.
	In chf, discontinue sunitinib or sorafenib, and start therapy for heart failure.
	Use sunitinib cautiously with drugs and conditions that prolong QT.
Interstitial pneumonitis	Radiographic changes without symptoms: continue temsirolimus and follow patient closely.
	In symptomatic interstitial pneumonitis, lung imaging and pulmonary functional tests are required. With mild symptoms, interrupt temsirolimus until symptoms resolve; with severe symptoms, discontinue temsirolimus and start treatment with oral high-dose steroids (for example, prednisone 1 mg/kg)
Laboratory toxicities	Hyperglycemia: fasting blood sugar should be tested before and during the treatment with temsirolimus; treat hyperglycemia with either or both of diet and oral agents (for example, metformin), or with insulin.
	Hyperlipidemia: serum cholesterol and triglycerides should be measured before and during treatment with temsirolimus. Use of statins may not be practical in this patient population. When triglycerides exceed 1000 mg/dL (11.3 mmol/L), consider treatment of hyperlipidemia or interrupt treatment with temsirolimus.
	Elevated serum lipase and amylase: serum lipase and amylase should be followed. Do not discontinue treatment with sunitinib or sorafenib if enzymes are elevated in the absence of clinical symptoms and signs of pancreatitis.
	Hypophosphatemia: serum phosphate levels should be measured regularly. In hypophosphatemia consider oral phosphate replacement (for example, sodium acid phosphate).
